# Prognostic Role of Nutritional and Inflammatory Indices in Predicting Adverse Clinical Outcomes in Unplanned Hospitalized Oncology Patients

**DOI:** 10.3390/jcm15082992

**Published:** 2026-04-15

**Authors:** Salih Karatlı, Doğan Yazılıtaş, Seher Kaya, Engin Yasin Baraklı, Selahattin Çelik, Gökşen İnanç İmamoğlu

**Affiliations:** 1 Department of Medical Oncology, Ankara Etlik City Hospital, 06710 Ankara, Türkiye; 2 Department of Internal Medicine, Ankara Etlik City Hospital, 06710 Ankara, Türkiye

**Keywords:** cancer, prognostic nutritional index, albumin-to-globulin ratio, intensive care, mortality, inflammation

## Abstract

**Background**: Unplanned hospitalizations in patients with cancer are associated with adverse outcomes, including intensive care unit (ICU) transfer and in-hospital mortality. This study aimed to evaluate the predictive role of the prognostic nutritional index (PNI) and albumin-to-globulin ratio (AGR) for these outcomes in patients with unplanned hospitalization in a medical oncology ward. **Methods**: This retrospective, single-center study included patients aged ≥18 years with malignancy who had unplanned hospitalization between 1 January and 30 April 2025. PNI and AGR were calculated at admission. The primary outcome was ICU transfer or in-hospital mortality. Univariable and multivariable logistic regression analyses were performed, with AGR and PNI evaluated in separate models to avoid collinearity. Predictive performance was assessed using ROC analysis. **Results**: A total of 418 patients were included, with adverse clinical outcomes in 26.8%. Metastatic disease was present in 73.7%, and gastrointestinal (41.6%) and lung cancers (21.5%) were most common. In univariable analysis, metastatic disease (*p* < 0.001), Eastern Cooperative Oncology Group (ECOG) performance status (*p* < 0.001), cancer type (*p* = 0.030), reason for hospitalization (*p* = 0.001), AGR (*p* < 0.001), and PNI (*p* < 0.001) were significantly associated with adverse clinical outcomes. In multivariable analyses performed in separate models, ECOG ≥ 2 emerged as the strongest independent predictor of adverse clinical outcomes (AGR model: OR: 9.93; PNI model: OR: 11.14; both *p* < 0.001). Metastatic disease remained an independent risk factor, while higher AGR and PNI values were independently associated with a reduced risk (all *p* < 0.05). Among hospitalization reasons, only electrolyte imbalance/transfusion was associated with a lower risk, whereas most cancer type subgroups were not independently significant. Both indices showed moderate predictive performance, with PNI performing slightly better than AGR (AUC: 0.729 vs. 0.707). **Conclusions**: ECOG performance status, together with PNI and AGR, were identified as practical and accessible predictors of adverse clinical outcomes in patients with unplanned hospitalization in a medical oncology ward.

## 1. Introduction

Cancer patients often require urgent medical evaluation due to complications such as disease progression, infection, or treatment-related toxicity, and many of these cases result in hospital admission. This subgroup represents a clinically vulnerable population with an increased likelihood of adverse outcomes, including transfer to the intensive care unit (ICU) and in-hospital death. Unplanned admissions, particularly in the setting of advanced-stage disease and high tumor burden, are consistently associated with unfavorable prognosis. Accordingly, early identification of high-risk patients is essential to optimize clinical decision-making and improve patient management [1,2,3,4].

Nutritional impairment and systemic inflammatory response are key determinants of clinical outcomes in oncology patients. The prognostic nutritional index (PNI), derived from serum albumin concentration and lymphocyte count, serves as a practical indicator of both nutritional and immune status and has been linked to survival, postoperative outcomes, and overall prognosis in various malignancies [5,6,7]. In parallel, the albumin-to-globulin ratio (AGR) represents a composite marker reflecting protein balance and inflammatory activity, with lower values consistently associated with poorer survival in patients with solid tumors [8,9,10].

In this study, we aimed to evaluate the predictive role of PNI and AGR for adverse clinical outcomes, defined as ICU transfer or in-hospital mortality, in patients with cancer undergoing unplanned hospitalization in a medical oncology ward following emergency department presentation. We hypothesized that these easily obtainable and cost-effective biomarkers could be useful for early risk stratification in this high-risk population and provide additional prognostic value in clinical practice.

## 2. Materials and Methods

### 2.1. Study Design and Patient Population

This study was designed as a retrospective, single-center observational study. Patients who were (unplanned) hospitalized in the medical oncology ward following emergency department presentation between 1 January 2025 and 30 April 2025 were evaluated.

#### 2.1.1. Inclusion Criteria

Patients meeting all of the following criteria were included:

- Age ≥ 18 years;
- Histologically or clinically confirmed diagnosis of malignancy;
- Unplanned hospitalization in the medical oncology ward following emergency department admission;
- Availability of complete laboratory data at admission (hemoglobin, lymphocyte count, platelet count, albumin, total protein, and globulin).

#### 2.1.2. Exclusion Criteria

- Age < 18 years;
- No confirmed diagnosis of malignancy;
- Missing laboratory data at admission;
- Elective (planned) hospitalizations.

### 2.2. Data Collection and Definitions

Demographic characteristics, comorbidities, cancer type, disease stage, Eastern Cooperative Oncology Group (ECOG) performance status, and reasons for hospitalization were retrospectively obtained from electronic medical records and the hospital information system.

Laboratory parameters at admission (hemoglobin, lymphocyte count, platelet count, albumin, total protein, and globulin) were recorded, and PNI and AGR were calculated based on these data. PNI was calculated using the formula: PNI = albumin (g/L) + 5 × lymphocyte count (×10^9^/L). AGR was calculated as AGR = albumin/(total protein − albumin).

The primary outcome was defined as adverse clinical outcome, including ICU transfer or in-hospital mortality during hospitalization. Although ICU transfer and in-hospital mortality represent distinct clinical outcomes, they were combined into a composite endpoint to better reflect real-world clinical practice and to increase statistical power.

### 2.3. Statistical Analysis

Statistical analyses were conducted using IBM SPSS Statistics version 25.0 (IBM Corp., Armonk, NY, USA). Continuous variables are presented as median values with corresponding ranges (minimum–maximum), while categorical variables are summarized as frequencies and percentages. Age was dichotomized based on the median value.

Univariable and multivariable logistic regression analyses were performed to identify factors associated with adverse clinical outcomes. Variables with a *p*-value < 0.10 in the univariable analysis and/or those considered clinically important based on prior literature and investigator judgment (such as metastatic disease and ECOG performance status) were included in the multivariable models. No stepwise selection was applied; all selected variables were entered simultaneously using the forced entry method.

Prior to multivariable modeling, multicollinearity among candidate predictors was formally assessed using variance inflation factors (VIFs) and tolerance statistics. A VIF > 5 or tolerance < 0.2 was considered indicative of multicollinearity. Because AGR and PNI share albumin as a common component, they were evaluated in separate multivariable models to avoid collinearity. All VIF values were <2, indicating no significant multicollinearity among the remaining variables.

Missing data were handled using a complete-case (listwise deletion) approach. A total of 49 patients were excluded from the analysis due to missing laboratory parameters required for the calculation of PNI and AGR; therefore, no imputation was performed.Model calibration was evaluated using the Hosmer–Lemeshow goodness-of-fit test, where a *p*-value greater than 0.05 was considered to indicate an acceptable model fit. Due to the retrospective single-center design, neither internal nor external validation of the models was performed, and this limitation is acknowledged in Section 4.

A *p*-value of <0.05 was considered statistically significant.

### 2.4. Ethical Approval

The study was approved by the Ethics Committee of Ankara Etlik City Hospital (Decision No: AEŞH-BADEK2-2025-399, 2 September 2025). Data extraction was performed after ethical approval. All procedures were conducted in accordance with the principles of the Declaration of Helsinki.

## 3. Results

A total of 418 patients were included in the study. The median age was 63 years (range: 20–90), and 58.4% of the patients were male. At least one comorbidity was present in 53.6% of the participants. The most common comorbidities were hypertension (33.5%) and diabetes mellitus (22.5%). The most frequent cancer types were gastrointestinal malignancies (41.6%) and lung cancer (21.5%). Overall, 73.7% of the patients had metastatic disease. Regarding performance status, 57.4% of the patients had ECOG 0–1, while 42.6% had ECOG ≥ 2. Adverse clinical outcomes (intensive care unit transfer or in-hospital mortality) were observed in 26.8% of the patients (Table 1).

At admission, the median hemoglobin level was 10.3 g/dL, lymphocyte count was 0.89 × 10^9^/L, albumin level was 33 g/L, and platelet count was 191 × 10^9^/L. The median PNI was 37.35, and the AGR was 1.23 (Table 2).

**Figure 1:**
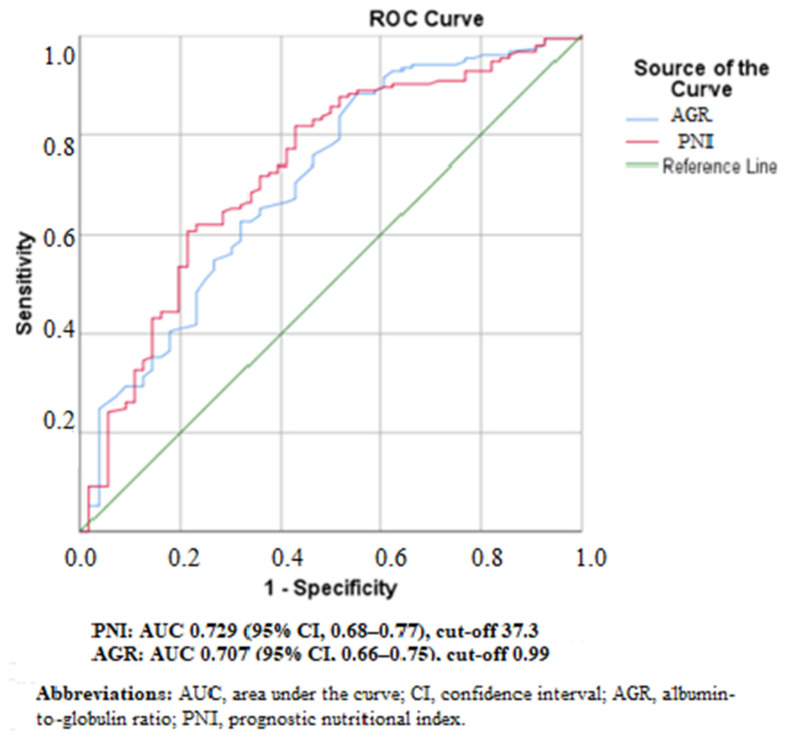
Receiver Operating Characteristic (ROC) Curves of AGR and PNI for Predicting Adverse Clinical Outcomes.

ROC analysis demonstrated that both AGR and PNI were significant predictors of adverse clinical outcomes. The AUC for PNI was 0.729 (95% CI: 0.68–0.77), and for AGR was 0.707 (95% CI: 0.66–0.75). PNI showed a slightly higher discriminative ability than AGR. The optimal cut-off values were determined as 0.99 for AGR (sensitivity 88.2%, specificity 55.4%) and 37.3 for PNI (sensitivity 60.8%, specificity 78.6%) (Figure 1).

In the univariable logistic regression analysis, metastatic disease was significantly associated with adverse clinical outcomes (OR: 3.93, 95% CI: 2.06–7.48, *p* < 0.001). ECOG performance status ≥ 2 was also strongly associated with an increased risk of adverse clinical outcomes (OR: 11.77, 95% CI: 6.83–20.27, *p* < 0.001). Cancer type and reason for hospitalization were significant in overall comparisons (*p* = 0.030 and *p* = 0.001, respectively). In subgroup analyses, patients admitted due to infection (OR: 0.41, 95% CI: 0.23–0.74, *p* = 0.003), interventional procedures (OR: 0.44, 95% CI: 0.24–0.80, *p* = 0.007), and electrolyte imbalance/transfusion (OR: 0.34, 95% CI: 0.18–0.64, *p* = 0.001) had a lower risk of adverse clinical outcomes compared to those admitted for supportive care and nutrition. Higher AGR and PNI values were found to be significantly protective against adverse clinical outcomes (OR: 0.17, 95% CI: 0.10–0.28 and OR: 0.18, 95% CI: 0.11–0.29, respectively; both *p* < 0.001). In contrast, age, sex, and comorbidity status were not significantly associated with adverse clinical outcomes (Table 3).

In the multivariable analysis, AGR and PNI were evaluated in separate models to avoid collinearity. ECOG performance status ≥ 2 emerged as the strongest independent predictor of adverse clinical outcomes in both models (AGR model: OR: 9.93, 95% CI: 5.49–17.93, *p* < 0.001; PNI model: OR: 11.14, 95% CI: 6.05–20.50, *p* < 0.001) (Table 4 and Table 5).

In the model including AGR, metastatic disease remained an independent risk factor (OR: 2.17, 95% CI: 1.02–4.63, *p* = 0.044). Among the reasons for hospitalization, only electrolyte imbalance/transfusion was associated with a lower risk compared to supportive care/nutrition (OR: 0.45, 95% CI: 0.21–0.97, *p* = 0.042). Higher AGR levels were independently associated with a reduced risk of adverse clinical outcomes (OR: 0.28, 95% CI: 0.15–0.51, *p* < 0.001). Cancer type was not independently associated with adverse clinical outcomes in this model (Table 4).

Similarly, in the model including PNI, metastatic disease remained an independent risk factor (OR: 2.32, 95% CI: 1.08–4.98, *p* = 0.031). Among cancer types, the “other” category was associated with a higher risk compared to lung cancer (OR: 2.40, 95% CI: 1.04–5.51, *p* = 0.040), whereas other subgroups were not significant. Electrolyte imbalance/transfusion was again associated with a lower risk (OR: 0.36, 95% CI: 0.17–0.80, *p* = 0.011). Higher PNI values were independently associated with a reduced risk of adverse clinical outcomes (OR: 0.19, 95% CI: 0.10–0.34, *p* < 0.001) (Table 5). The Hosmer–Lemeshow test demonstrated good model calibration for both models (AGR model: *p* = 0.41; PNI model: *p* = 0.52).

## 4. Discussion

In this study, adverse clinical outcomes—including ICU transfer and in-hospital mortality—were common among patients with unplanned oncology admissions. Our findings demonstrate that PNI and AGR are independent and clinically meaningful predictors of these outcomes. Among all evaluated variables, ECOG performance status emerged as the strongest determinant, while metastatic disease was also independently associated with an increased risk. These findings support the clinical utility of simple and readily available biomarkers, such as PNI and AGR, for early risk stratification in this high-risk patient population.

In our previous study, conducted in a 2024 cohort, we evaluated the prognostic value of the Hemoglobin, Albumin, Lymphocyte, and Platelet (HALP) score and the Modified Glasgow Prognostic Score (mGPS) in predicting ICU requirement among solid tumor patients hospitalized in the medical oncology ward for various clinical reasons, excluding elective admissions solely for active treatment administration [11]. In contrast, the present study expands upon this work by analyzing a distinct patient population from 2025, with a specific focus on unplanned hospitalizations and a broader composite endpoint of adverse clinical outcomes (ICU transfer and in-hospital mortality), while also investigating alternative inflammation- and nutrition-based indices, namely the PNI and the AGR. These methodological and conceptual differences indicate that the present study represents a distinct and non-overlapping contribution to the literature.

Extensive research indicates that unplanned ICU admissions for cancer patients are a strong predictor of poor clinical outcomes. Reported in-hospital mortality rates for this cohort vary significantly, ranging from 11% to 58%. These high mortality rates are primarily attributed to advanced malignancy, substantial tumor burden, and pre-existing comorbidities, further exacerbated by delays in initiating early supportive interventions [1,12,13]. The rate of adverse clinical outcomes observed in our study appears to be consistent with previously reported unplanned oncology cohorts.

Metastatic disease represents a complex clinical state characterized by increased tumor burden, reduced organ reserve, immune dysregulation, and greater vulnerability to treatment-related toxicities. These elements collectively heighten the risk of serious complications, including sepsis, multi-organ failure, and the need for intensive care unit admission. In line with previous studies, our multivariable analysis confirmed metastatic disease as an independent predictor of adverse clinical outcomes in patients with unplanned oncology ward admissions [14,15]. While metastatic involvement maintained its independent prognostic significance in our multivariable models, its effect size was attenuated when ECOG performance status was included. Such a shift indicates that the relationship between metastatic stage and unfavorable outcomes is likely influenced by the patient’s functional baseline. Consequently, these findings underscore the importance of functional reserve—as captured by ECOG PS—as a pivotal mediator that translates disease burden into clinical prognosis.

Our analysis identified ECOG performance status as the preeminent predictor of unfavorable clinical trajectories, highlighting that a patient’s functional reserve is central to their survival outlook during hospitalization. Consistent with established evidence, these results confirm that performance status remains a pivotal metric for evaluating not only life expectancy but also the patient’s resilience against treatment-related toxicities and clinical deterioration [16,17,18,19]. The strong effect size observed in our analysis further emphasizes that functional impairment may outweigh other clinical factors, including disease stage, in predicting short-term outcomes.

In oncology patients, ICU admission is most commonly driven by infections, respiratory failure, and multi-organ dysfunction, particularly in the setting of unplanned hospitalizations [16,17,18,19]. In our study, patients with adverse clinical outcomes were more likely to have advanced disease and require supportive care, whereas those admitted for reversible conditions such as electrolyte imbalance or transfusion had a lower risk.

PNI and AGR were identified as independent protective factors, highlighting the impact of nutritional status and systemic inflammation on clinical outcomes. While PNI reflects both immune competence and nutritional reserve, AGR represents the balance between protein status and inflammatory burden. Lower values of these indices may indicate increased vulnerability to clinical deterioration. These findings are consistent with previous studies demonstrating the prognostic value of PNI and AGR across different cancer populations [20,21,22,23,24,25].

The slightly higher discriminative performance of PNI compared to AGR in ROC analysis suggests that an index incorporating both nutritional and immune parameters may provide superior predictive value. This may be explained by the inclusion of lymphocyte count in PNI, reflecting cellular immune function and overall physiological reserve. In contrast, AGR primarily reflects protein balance and inflammatory status and may not directly capture immune competence. Nevertheless, both indices are easily obtainable from routine laboratory data without additional cost, making them practical tools for early risk stratification in this high-risk population.

This study has several limitations. Its retrospective and single-center design may introduce selection bias and limit generalizability. The use of only admission laboratory values precludes assessment of dynamic changes over time. In addition, continuous variables were dichotomized to enhance clinical interpretability, which may have led to loss of information and reduced statistical power. The heterogeneity of cancer types may also influence the results, and certain clinical variables, such as treatment-related factors (e.g., chemotherapy, immunotherapy), detailed measures of disease burden beyond metastatic status, severity-of-illness scores (e.g., Sequential Organ Failure Assessment (SOFA) and Acute Physiology and Chronic Health Evaluation II (APACHE II)), and molecular characteristics, were not included in the analyses due to data limitations, which may have led to residual confounding. In our clinical practice, patients who experience clinical deterioration are rapidly transferred to the ICU, which may explain the relatively low number of in-hospital deaths observed in the oncology ward (*n* = 20). Therefore, ICU transfer and in-hospital mortality were combined as a composite endpoint. While this approach increases statistical power, it may also obscure differences between these two clinically distinct endpoints. Therefore, the results should be interpreted with consideration of this potential heterogeneity. Moreover, the study period did not cover a full year, and seasonal or temporal variations may have influenced the findings. Long-term outcomes such as 90-day mortality or overall survival were not evaluated. Furthermore, the absence of external validation limits the generalizability of the identified cut-off values and the predictive performance of the models. In addition, neither internal nor external validation of the predictive models was performed, which may limit the robustness and reproducibility of the findings, and the possibility of model overfitting cannot be excluded.

## 5. Conclusions

ECOG performance status, PNI, and AGR were identified as independent, accessible, and cost-effective predictors of adverse clinical outcomes in patients with cancer who are (unplanned) hospitalized in a medical oncology ward. Integration of these indices into routine clinical assessment may facilitate early identification of high-risk patients and improve clinical decision-making and patient management. Future prospective multicenter studies are warranted to validate these findings.

## Figures and Tables

**Table 1:** Clinical and Demographic Characteristics of the Study Population.

Variable	*n* (%)
Total patients	418
Age, median (range)	63 (20–90)
Male	244 (58.4%)
Female	174 (41.6%)
Any comorbidity	224 (53.6%)
Hypertension	140 (33.5%)
Diabetes mellitus	94 (22.5%)
Cardiovascular disease	70 (16.7%)
Respiratory disease	44 (10.5%)
Thyroid disease	24 (5.7%)
Neurological disease	34 (8.1%)
Lung cancer	90 (21.5%)
GI malignancies	174 (41.6%)
Breast cancer	42 (10.0%)
Genitourinary cancers	28 (6.7%)
Gynecological cancers	28 (6.7%)
Head and neck cancers	32 (7.7%)
Others	24 (5.7%)
Non-metastatic	110 (26.3%)
ECOG 0–1	240 (57.4%)
ECOG ≥ 2	178 (42.6%)
Adverse clinical outcome (ICU transfer or in-hospital mortality)	112 (26.8%)

Abbreviations: GI: Gastrointestinal; ICU: Intensive Care Unit; ECOG: Eastern Cooperative Oncology Group.

**Table 2:** Laboratory Parameters and Nutritional–Inflammatory Indices at Admission.

Parameter	Median (Min–Max)
Hemoglobin (g/dL)	10.3 (5.3–17.8)
Lymphocyte (×10^9^/L)	0.89 (0.06–5.57)
Albumin (g/L)	33 (14–48)
Total protein (g/L)	60 (35–80)
Platelet (×10^9^/L)	191 (6–1750)
Globulin (g/L)	27 (12–65)
PNI	37.35 (19.6–63.85)
AGR	1.23 (0.48–3.17)

Abbreviations: PNI: Prognostic Nutritional Index; AGR: albumin-to-globulin ratio.

**Table 3:** Univariable Logistic Regression Analysis for Predicting Adverse Clinical Outcomes (ICU Transfer or In-Hospital Mortality).

Variable	OR (Exp(B))	95% CI	*p*-Value
Sex (Male)	0.76	0.49–1.18	0.229
Age (≥63 vs. <63)	0.90	0.58–1.39	0.639
Comorbidity (present)	0.91	0.59–1.39	0.655
Metastatic disease	3.93	2.06–7.48	<0.001
ECOG ≥ 2 vs. ECOG 0–1	11.77	6.83–20.27	<0.001
Cancer type (overall)	—	—	0.030
GI vs. lung	2.00	1.09–3.66	0.025
Breast vs. lung	0.67	0.24–1.82	0.430
Others vs. lung	1.46	0.75–2.84	0.261
Reason for hospitalization (overall)	—	—	0.001
Infection vs. supportive care/nutrition	0.41	0.23–0.74	0.003
Interventional causes vs. supportive care/nutrition	0.44	0.24–0.80	0.007
Electrolyte imbalance/transfusion vs. supportive care/nutrition	0.34	0.18–0.64	0.001
AGR (high vs. low)	0.17	0.10–0.28	<0.001
PNI (high vs. low)	0.18	0.11–0.29	<0.001

Abbreviations: OR: Odds Ratio; CI: Confidence Interval; ICU: Intensive Care Unit; AGR: albumin-to-globulin ratio; PNI: Prognostic Nutritional Index; GI: Gastrointestinal; ECOG: Eastern Cooperative Oncology Group.

**Table 4:** Multivariable Logistic Regression Analysis Including AGR for Predicting Adverse Clinical Outcomes (ICU Transfer or In-Hospital Mortality).

Variable	OR (Exp(B))	95% CI	*p*-Value
ECOG ≥ 2 vs. ECOG 0–1	9.93	5.43–17.93	<0.001
Metastatic disease	2.17	1.02–4.63	0.044
Cancer type (overall)	—	—	0.065
GI vs. lung	1.18	0.56–2.46	0.667
Breast vs. lung	0.37	0.11–1.20	0.097
Others vs. lung	1.71	0.77–3.78	0.186
Reason for hospitalization (overall)	—	—	0.166
Infection vs. supportive care/nutrition	1.07	0.51–2.23	0.864
Interventional causes vs. supportive care/nutrition	0.80	0.38–1.67	0.548
Electrolyte imbalance/transfusion vs. supportive care/nutrition	0.45	0.21–0.97	0.042
AGR (high vs. low)	0.28	0.15–0.51	<0.001

Abbreviations: OR: Odds Ratio; CI: Confidence Interval; ICU: Intensive Care Unit; AGR: albumin-to-globulin ratio; PNI: Prognostic Nutritional Index; GI: Gastrointestinal; ECOG: Eastern Cooperative Oncology Group.

**Table 5:** Multivariable Logistic Regression Analysis Including PNI for Predicting Adverse Clinical Outcomes (ICU Transfer or In-Hospital Mortality).

Variable	OR (Exp(B))	95% CI	*p*-Value
ECOG ≥ 2 vs. ECOG 0–1	11.14	6.05–20.50	<0.001
Metastatic disease	2.32	1.08–4.98	0.031
Cancer type (overall)	—	—	0.09
GI vs. lung	1.23	0.58–2.60	0.597
Breast vs. lung	0.32	0.10–1.08	0.067
Others vs. lung	2.40	1.04–5.51	0.040
Reason for hospitalization (overall)	—	—	0.071
Infection vs. supportive care/nutrition	0.96	0.45–2.04	0.915
Interventional causes vs. supportive care/nutrition	0.84	0.39–1.78	0.645
Electrolyte imbalance/transfusion vs. supportive care/nutrition	0.36	0.17–0.80	0.011
PNI (high vs. low)	0.19	0.10–0.34	<0.001

Abbreviations: OR: Odds Ratio; CI: Confidence Interval; ICU: Intensive Care Unit; AGR: albumin-to-globulin ratio; PNI: Prognostic Nutritional Index; GI: Gastrointestinal; ECOG: Eastern Cooperative Oncology Group.

## Data Availability

The datasets generated and analyzed during the current study are available from the corresponding author upon reasonable request.

## Abbreviations

The following abbreviations are used in this manuscript:

AGR	albumin-to-globulin ratio
AUC	area under the curve
CI	confidence interval
ECOG	Eastern Cooperative Oncology Group
GI	gastrointestinal
ICU	intensive care unit
OR	odds ratio
PNI	prognostic nutritional index
ROC	receiver operating characteristic
